# The nightjar and the ant: Intercontinental migration reveals a cryptic interaction

**DOI:** 10.1002/ece3.11113

**Published:** 2024-05-20

**Authors:** Carlos Camacho, J. Manuel Vidal‐Cordero, Pedro Sáez‐Gómez, Paula Hidalgo‐Rodríguez, Julio Rabadán‐González, Carlos Molina, Pim Edelaar

**Affiliations:** ^1^ Department of Ecology and Evolution Estación Biológica de Doñana–CSIC Sevilla Spain; ^2^ Department of Ecology, Terrestrial Ecology Group (TEG‐UAM) Universidad Autónoma de Madrid Madrid Spain; ^3^ Department of Molecular Biology and Biochemical Engineering Universidad Pablo de Olavide Sevilla Spain; ^4^ Observation.org Sevilla Spain; ^5^ SEO/BirdLife, Doñana Technical Office El Rocío, Huelva Spain

**Keywords:** amputations, *Caprimulgus*, *Dorylus*, interspecific interactions, migration

## Abstract

Birds and ants co‐occur in most terrestrial ecosystems and engage in a range of interactions. Competition, mutualism and predation are prominent examples of these interactions, but there are possibly many others that remain to be identified and characterized. This study provides quantitative estimates of the frequency of toe amputations resulting from ant bites in a population of migratory red‐necked nightjars (*Caprimulgus ruficollis*) monitored for 15 years (2009–2023) in S Spain, and identifies the attacker(s) based on taxonomic analyses of ant‐mandible remains found on injured toes. Less than 1% of examined adults (*N* = 369) missed one or more toes. The analysis of ant remains identified African army ants (*Dorylus* sp.) as the primary cause of toe amputations in nightjars and revealed that body parts of the attacker may remain attached to the birds even after intercontinental migration. No cases of severe damage were observed in juveniles (*N* = 269), apart from the mandible of a *Messor barbarus* – a local ant species – attached to one of the teeth of the characteristic comb of the medial toe of nightjars. The incidence of ant‐bite damage may appear unimportant for nightjar populations, but this might not be true if only birds that manage to survive their injuries and potential complications (e.g. severe bleeding and sepsis from opportunistic infections) return from the tropics. More field studies, ideally in tropical areas, that incorporate routine examination of ant‐induced injuries into their protocols are needed to understand the true incidence and eco‐evolutionary implications of antagonistic ant‐bird interactions.

## Graphical abstract

Nightjars are migratory birds that spend most of their time on the ground and are exposed to accidental contact with army ants on their tropical non‐breeding grounds. Damage from ant bites results in toe amputations in at least 1% of individuals. Mandible remains of the ants may still be found attached to the toes of nightjars breeding in Europe even after migration from tropical areas, enabling determination of the cause of injury and identification of the attacker.

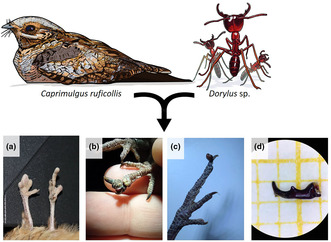

## 1 INTRODUCTION

The interactions between birds and insects have been the subject of extensive research (Morse, 1971; Willis & Oniki, 1978). Bird‐insect interactions in the form of predation, parasitism, and mutualism have received the most attention because of their ubiquitous occurrence and importance as study systems for understanding coevolution (Holmes et al., 1979; Maziarz et al., 2021; Price, 1997; Quinn & Ueta, 2008). Other less conventional or more cryptic interaction types also occur along the antagonism‐commensalism gradient, such as competition (e.g. for food resources; Smith & Balda, 1979) or ‘anting’ (i.e. the use of ants as a treatment against parasites; Camacho & Potti, 2018), but they often go undetected or underreported and their true nature and biological significance remain to be elucidated in most cases.

Of all insects, ants (Formicidae) are the most abundant in most terrestrial ecosystems (Del Toro et al., 2012). There is ample literature documenting antagonistic ant‐bird interactions, primarily in the form of bird predation on ants (reviewed in Avilés, 2024). There are also studies reporting negative effects of ants on aspects such as bird abundance, behaviour and reproduction, primarily through predation on eggs and chicks (Álvarez‐Blanco et al., 2020; Davis et al., 2008; Haemig., 1999). However, assessing the impact of ants on more mobile segments of a population (e.g. juveniles and adults) is challenging and our understanding of the nature and magnitude of these effects is therefore limited.

This study takes advantage of a long‐term capture‐recapture program to examine the nature and frequency of injuries (toe amputations) of a migratory insectivorous bird – the red‐necked nightjar (*Caprimulgus ruficollis*) – in their breeding area in S Spain. The study was motivated by observations of nightjars missing toes, particularly of an adult bird recorded in 2015 that had what appeared to be the mandible of an ant embedded in the medial toe of one of their feet. Six years later, in 2021, we found another adult that had the full head of an ant embedded in a central toe. Since then, we engaged in a systematic revision of the feet of all captured nightjars to evaluate the frequency and severity of toe injuries. Observations of insect remains attached to injured toes in three nightjars provided an opportunity to identify the agents of damage based on a taxonomic analysis. We close by discussing the incidence of such injuries on nightjar populations and identify further research needs.

## 2 METHODS

### 2.1 Study system

Data on toe damage were collected during three breeding seasons (2021–2023) as part of a long‐term (2009‐present) study of red‐necked nightjars in the Doñana National Park, S Spain (see Camacho et al., 2014, 2022 for details on the study site and sampling protocols). Most nightjars arrive in the study area in early May and generally postpone their migration departure until mid‐October (i.e. approx. 5½‐month stay in the breeding area; Camacho, 2013a). GPS tracks of adult nightjars from the study population indicate that most individuals migrate through Morocco and Mauritania to spend the non‐breeding season (November–April, approx. 5½ months) in S Mali and SE Guinea (C. Camacho and P. Sáez‐Gómez, unpublished data).

Nightjars spend much of their time on the ground: they are ground‐nesting birds and both members of the pair share incubation and chick‐rearing duties for over 35 days, the female taking the larger share (Camacho, 2013a). During the day, they remain motionless on the ground in the shade of trees or shrubs (Camacho et al., 2014). During the night, they use open spaces on the ground as perches to facilitate detection of aerial insects (mainly moths), although they can also occasionally pick up terrestrial insects that are running around (Jackson, 2003). In spending so much time on the ground, nightjars are exposed to accidental contact with all sorts of diurnal and nocturnal ground‐dwelling creatures.

### 2.2 Field and lab procedures

Nightjars are attracted to roads at night (Jackson, 2003), so road transects are one of the most commonly used methods for nightjar monitoring (Camacho, 2013b; De Felipe et al., 2019). From late March to late October, we conducted nocturnal surveys for red‐necked nightjars on a regular (3–7 days) basis along a 24‐km road transect by driving a car at slow speed (30 km h^−1^). Nightjars found on roads were captured using a torch and a butterfly net (Jackson, 1984) and marked with numbered metal rings (if not ringed already) for individual recognition. Birds were sexed and aged as first‐year (juvenile), second‐year (approx. 1 year old), or older using plumage characteristics (Camacho, 2013a; Forero et al., 1995), and measured according to the standard protocol for long‐term monitoring of nightjars (see details in Hidalgo‐Rodríguez et al., 2021). Many individuals' ages are known because they were first captured as juveniles (Camacho et al., 2022).

**FIGURE 1:**
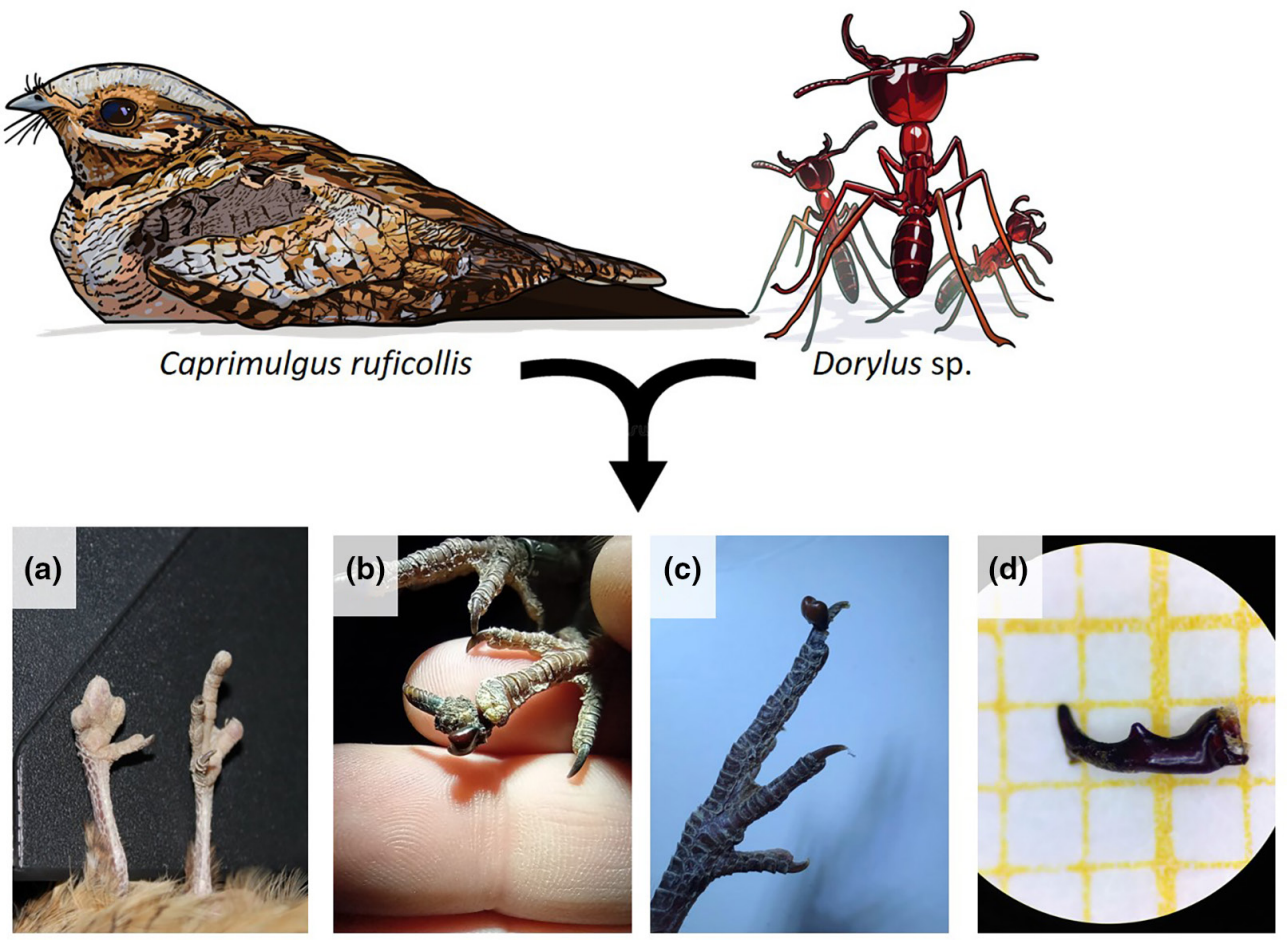
Observed ant‐induced injuries on Nightjars (a–c), and detail of *Dorylus* sp. mandible (d) collected from one of them. Note that image (c) does not correspond to a *Dorylus* ant, but to a *Messor barbarus* found on a juvenile nightjar.

To estimate the frequency of damage on limbs, we carefully examined the feet and toes of all captured individuals, noted anomalies (e.g. breaks in skin, and full or partial amputations; Figure 1a) and checked for remains of the attacker (e.g. mandibles or full head; Figure 1b–d). The remains found during the 3 years of systematic record were removed from the bird and preserved in 96° ethanol for taxonomic identification in the laboratory. The remains, specifically a left mandible found in 2021 and a full head found in 2023, were closely examined under an 80× stereomicroscope to identify the attacker to the genus or species level (Borowiec, 2016; Fisher & Bolton, 2016).

We calculated the proportion of juveniles and adults missing feet or toes (or in the process of losing them, as determined from the severity of damage) as a measure of the incidence of damage, and used the Fisher's exact test as implemented in R (https://cran.r‐project.org/) to test the null hypothesis that the incidence of damage does not differ between age classes. Non‐systematic data collected from birds trapped during ringing operations in previous years (2009–2020) provided additional useful information about the nature of damage.

## 3 RESULTS

Our dataset consisted of a total of 855 captures from 588 individual nightjars that had been examined for limb damage and loss at least once during 97 nocturnal surveys conducted in 2021–2023 (Table 1). Sampling effort, measured as either the total number of captures (range: 282–287) or as the number of different individuals examined (range: 207–225), was uniform across the 3 years of systematic study (Table 1). No evidence of severe damage (i.e. toe amputation) was observed in any of the 219 juveniles examined, although one of the juveniles caught in 2023 had a full (ant) head attached to the claw of the central toe (Figure 1c). Of the 369 different adults captured during 2021–2023, only three missed a toe: a 1‐year‐old male captured for the first time in 2021 (toe still attached to the foot by a thin piece of skin; Figure 1b), a 5‐year‐old male captured in both 2021 and 2022 (first caught as a second‐year bird in 2017), and a 1‐year‐old female captured in 2023 (first caught as a non‐injured juvenile the year before). The estimated frequency of damage in adults across the 3 years of study was 1:123 (0.81%), although annual estimates differed as much as 2‐fold (Table 1). Four additional adults missing one or more phalanges in one or more toes were recorded during non‐systematic checks in previous years (Table 1). Despite the lack of records of toe amputations in juvenile nightjars, there is insufficient statistical support to conclude that there is a greater incidence of damage in adults (Fisher's exact test, *p* = .299).

**TABLE 1:** Number of captures, number of individuals examined, and proportion of injured birds recorded during the 3‐year period of systematic study and the previous 12 years.

	Sampling effort (captures)	No. adults	No. juveniles	No. injured adults (%)	No. injured juveniles (%)
Systematic study					
2023	286	134	73	1 (0.75)	0 (0)
2022	287	160	65	1 (0.63)	0 (0)
2021	282	139	81	2 (1.44)	0 (0)
2021–2023	855	369	219	3 (0.81)	0 (0)
Non‐systematic study					
2009–2020	3554	1099	926	4 (0.4)	0 (0)

*Note*: Since bird‐ant interactions can occur at any time, individuals are included for as many years as they have been checked. Note that for this reason the summation of annual numbers of recorded adults does not match the grand total of different adults recorded in 2021–2023.

Damage generally consisted of total amputation (80% of cases) or incomplete excision (20%) of one or two toes. The most severe damage was recorded for an adult female captured in 2015, which missed three toes of the right foot and one lateral toe and the claw of the medial toe on the left foot (Figure 1a).

The encountered left mandible (year 2021) measured 2.3 mm in length by a maximum width of 0.8 mm, was dark brown in colour and ended in a bifurcation, with a robust central tooth. It was identified as belonging to a worker ant of the army ant genus *Dorylus* (Fabricius, 1793). Some 20 species of *Dorylus* ants have been listed for Mali and Guinea (antsmap.org), where the captured nightjars spend the winter. It is currently impossible to identify the attacker at the species level, even if we had retrieved the entire individual, and the entire genus is currently under taxonomic revision (Kiko Gómez, personal communication).

The full head attached to the juvenile (year 2023) caused no apparent damage to the bird, apart from nicking one of the teeth of the characteristic pecten (comb) of the medial toe of nightjars (Figure 1c). The full head was identified as belonging to a worker of *Messor barbarus*, an easily identifiable species that can be distinguished from other species of the genus in the Iberian Peninsula by the unique reddish colouration on the head (Lebas et al., 2017).

## 4 DISCUSSION

We provided quantitative estimates of the frequency of toe amputations in juvenile and adult red‐necked nightjars, evaluated the severity of damage to the birds, and identified the body parts of the ants that remained attached to nightjar toes. Less than <1% of individuals missed one or more toes. Toe amputations were only observed in adults that had performed at least one round‐trip migration from Europe to Africa and, although ant‐nightjar encounters also occurred in juveniles, these resulted in minor injuries. The taxonomic analysis of mandible remains identified army‐ant bites as the most likely cause of toe amputations in adult nightjars.

Our estimate of the incidence of ant bites is based on the proportion of toe‐mutilated birds, but the cause of damage could only be confirmed for those individuals that had remains of ant mandibles or the full head on their toes. None of the nightjars examined in this population (>4500 captures) exhibited typical poxvirus‐like lesions on the eyes, feet or toes, suggesting that poxvirus infection does not play a major role in the observed toe amputations. Besides amputations, over the 15 years of study we have observed structural injuries to the sternum (*n* = 7), tarsus (*n* = 1), mandibles (*n* = 4), and toes (*n* = 5) of nightjars, affecting <1% of all trapped individuals. These injuries are hypothesized to reflect healed fractures based on the presence of bone callus formation and/or angular deformity. Damaged toes due to traumatic fracture differ from confirmed ant‐bitten toes in that fractured toes often become distorted in position, pointing obliquely across the other toes, but the skin remains intact. By contrast, army ant bites result in a break in the skin of the affected phalanx and, even though the compressive force applied to the toe is not enough to fracture the bone, prolonged compression might cause ischemia and subsequent necrosis of the bone tissue, ultimately resulting in the excision and loss of one or more of the phalanges or the entire toe. The anatomical similarities of mutilations among birds missing toes and the confirmed ant‐bite injuries in birds in the process of losing a toe suggest that ant bites, rather than laceration from trauma (e.g. road traffic or building collisions) or predator attacks (e.g. carnivores), are the most likely cause of the observed toe amputations in adult nightjars.

No trace of severe damage from ant bites was seen in juveniles despite their sample size being even larger than that of adults (1145 juveniles vs. 1104 adults considering all years of the long‐term study). Nevertheless, the total number of injured birds is too small to support the statement that injuries from ant bites are restricted to adults. The observation of the full head of a *M. barbarus* attached to the pecten of the medial toe of a juvenile demonstrates that they are not entirely free from ant attacks, although ant species occurring in south Spain are likely too small for their mandibles to grasp on the relatively thick toes of a nightjar. *M. barbarus* is very common on the Mediterranean coast (Lebas et al., 2017), but it may not pose as much of a threat to nightjars as army ants because *M. barbarus* feed mainly on seeds and their mandibles are relatively small in comparison to those of army ants.

The genus *Dorylus* ranges from sub‐Saharan Africa across North Africa and Asia Minor to Borneo in Southeast Asia. The Afrotropics harbour the largest number of species (116 so far described), including subterranean‐, leaf litter‐ and surface‐dwelling species. Of these, surface‐dwelling species have, among other characteristics, the greatest mandible length and mandibular aperture (Schöning et al., 2005). *Dorylus* species are generalist predators that take any type of prey, from immature individuals of other insects to vertebrate carrion (Schöning & Moffett, 2007). Unlike leaf litter‐dwelling and subterranean species, *Dorylus* ants spend much of their time foraging on the soil surface, and given their mobile, predatory lifestyle and aggressive behaviour, attacks on the birds they encounter on the ground should be common.

Nineteenth‐century naturalists assumed that birds eat army ants but, as evidence accumulated about the foraging patterns of birds, it became clear that birds peck only at ants attacking their feet or plumage (Willis & Oniki, 1978). No mention of army ants is made in the published diet studies of Afrotropical nightjars (Jackson, 2000). In addition, although army ants are major predators of vertebrates (e.g. birds, sea turtles), most documented cases of predation on birds took place during the nesting stage (Ikaran et al., 2020; Robinson & Robinson, 2001). None of these organisms therefore appear to serve as a trophic resource for the other, suggesting that ant‐nightjars encounters are coincidental and detrimental to at least one and possibly both parties.

Missing and damaged toes have also been observed in other migratory and sedentary bird species and, as in the red‐necked nightjar, these injuries were hypothesized to result from ant attacks (e.g. Ibáñez et al., 2007; Sugg, 1974). Sugg (1974) reported on the specific bite effects by *Dorylus* sp. on 10 out of 343 (2.9%) pied kingfishers (*Ceryle rudis*) from a breeding colony on the Kenyan shore of Lake Victoria. Toe amputations and damage from ant bites appear to be more common in pied kingfishers (3%) than in red‐necked nightjars (<1%) despite the latter spending more time on the ground. The estimated proportion of toe amputations in another population of red‐necked nightjars monitored for 6 years in E Spain is 0.4% (three cases out of 751 different adults examined in 2017–2022; J.M. Zamora, personal communication). The field protocols in this study, as in the first years of our long‐term study, did not include routine screening for toe or foot anomalies, so estimates are based on opportunistic observations made during ringing sessions. The proportions of toe amputations in both populations, as estimated from opportunistic observations, are identical (0.4%) and half the magnitude of that based on a systematic examination of *both* feet (0.8%), suggesting that routine screening for anomalies is needed to obtain unbiased estimates. Caution is therefore required in extrapolating our results to other populations, species and geographic regions. Different estimates could be obtained if we study nightjars in their wintering area (where ants occur). The frequency of ant‐bite damage in breeding nightjars appears almost negligible. However, this might be an underestimate if only birds that manage to survive their injuries migrate successfully from their non‐breeding grounds to our study area.

Most injuries observed on nightjars, as in the pied kingfishers, consisted of single‐toe amputations that should not necessarily impair the birds' functional capacities and compromise performance in tasks such as predator avoidance or food acquisition. One of the injured nightjars actually survived from one breeding season to the next. Nonetheless, the loss of the medial toe and, therefore, of the pecten could be detrimental to nightjars because, even though the exact purpose of the pecten remains unclear, it has been suggested that it might be used as a comb for removing parasites during preening, or to straighten out the rictal bristles (Cleere, 2010). Moreover, as evidenced by at least one of the injured nightjars, multiple ant bites on the same bird might cause multiple amputations, potentially compromising locomotion on a chronic basis and, ultimately, long‐term survival. Breaks in the skin from ant bites, even if affecting only one toe, might increase the risk of fatal complications, such as severe bleeding and sepsis from opportunistic infections, so some individuals may die before migrating and being captured on their breeding grounds. That is, only the fraction of birds that survive ant attacks and subsequent damage might be recorded by us. Hence, the true incidence of ant bites on birds could be much higher than it appears to be.

Further research is required to understand the true incidence and eco‐evolutionary implications of antagonistic ant‐bird interactions. Being accidental, the factors promoting ant‐nightjar encounters are difficult to determine, and consequences different from injuries (e.g. a change in behaviour, systemic symptoms) are difficult to assess and require more detailed study. Fortunately, migratory nightjars are becoming popular model organisms in movement ecology studies across the globe, including capture programs conducted in tropical areas during the non‐breeding season (e.g. Cockle et al., 2023; Evens et al., 2023). Thus, it is possible that studies similar to ours could be attempted in other populations, species and geographic regions. More of these studies should encompass areas of the distribution range of large, tropical ants and incorporate routine examination and systematic recording of injuries into their protocols to increase the chances of encountering clear evidence of the agent of damage and to obtain reliable estimates of the true frequency of injuries and their origin (stochastic or not). Moreover, studies that consider aspects of performance (e.g. mating success, foraging success) and components of fitness (e.g. survival, reproductive success or lifespan) in injured birds relative to uninjured ones are encouraged as an essential step to understand the influence of injuries on natural selection and evolution (Hendry et al., 2022).

## AUTHOR CONTRIBUTIONS

**Carlos Camacho:** Conceptualization (lead); data curation (lead); formal analysis (lead); funding acquisition (supporting); investigation (equal); methodology (equal); project administration (lead); visualization (supporting); writing – original draft (lead); writing – review and editing (equal). **J. Manuel Vidal‐Cordero:** Conceptualization (equal); formal analysis (equal); investigation (equal); methodology (equal); visualization (lead); writing – original draft (supporting); writing – review and editing (equal). **Pedro Sáez‐Gómez:** Conceptualization (equal); data curation (equal); investigation (equal); methodology (equal); writing – review and editing (equal). **Julio Rabadán‐González:** Conceptualization (equal); data curation (lead); investigation (equal); methodology (equal); writing – review and editing (equal). **Paula Hidalgo‐Rodríguez:** Conceptualization (equal); data curation (equal); investigation (equal); methodology (equal); project administration (supporting); writing – review and editing (equal). **Carlos Molina:** Conceptualization (equal); data curation (equal); investigation (equal); methodology (equal); writing – review and editing (equal). **Pim Edelaar:** Conceptualization (equal); funding acquisition (lead); investigation (equal); methodology (equal); project administration (lead); writing – original draft (supporting); writing – review and editing (equal).

## CONFLICT OF INTEREST STATEMENT

The authors declare that there are no conflicts of interest related to this study.

## Data Availability

The data used in this study are presented in Table 1.
